# Arthroscopic Repair of Rotator Cuff Tears in Older Adults: A Retrospective Case-Series Study

**DOI:** 10.1177/21514593241294045

**Published:** 2024-10-15

**Authors:** Danyal Baytoon, Viktor Schmidt, Aleksander Bazan, Mats Wadsten, Arkan Sayed-Noor

**Affiliations:** 1 Department of Diagnostics and Intervention (Orthopedics), 8075 Umeå University, Umea, Sweden; 2 College of Medicine, 105977 University of Sharjah, Sharjah, United Arab Emirates

**Keywords:** sports medicine, upper extremity surgery, rotator cuff tear, geriatric trauma, arthroscopic rotator cuff repair

## Abstract

**Introduction:**

Rotator cuff tears (RCTs) are a common source of pain in the shoulder girdle. There is still debate about the optimal treatment for older adults with RCTs. In clinical practice, patients who do not respond well to non-surgical management may still be eligible for operative treatment. In this study, we assessed the outcome of arthroscopic repair of RCTs in patients ≥60 years old.

**Material and method:**

A retrospective case series was conducted to include patients who underwent arthroscopic repair of RCTs from 1 January 2018 to 1 January 2021. The study included individuals aged ≥60 years who had radiologically confirmed RCTs (verified by MRI) and clinical findings including sleep-disturbing pain and reduced range of motion. Preoperative treatment included physiotherapy for at least 6 months and one subacromial corticosteroid injection.

**Results:**

Fifty-three RCTs were treated during the study period. After exclusion because of incomplete documentation, 45 patients remained. The mean age was 66 years and 80% had isolated supraspinatus tears and 25% had variable degrees of fatty infiltration (Goutallier grade 1-3) on MRI examination with positive tangent sign. There were no surgical site infections and three symptomatic re-ruptures (6%). At follow-up, (71%) reported no remaining sleep-disturbing shoulder pain. Abduction improved from 62° to 122°. Flexion improved from 68° to 135°. This study found that people aged 60 years and older who underwent repair of RCTs showed statistically significant clinical improvement in shoulder flexion and abduction with less sleep-disturbing shoulder pain. These encouraging results may contribute to the existing literature, favoring the choice of surgical treatment for symptomatic RCTs in this age group with failed nonoperative treatment.

**Conclusion:**

The arthroscopic repair of RCTs in patients 60 years and older yielded a substantial increase in shoulder flexion and abduction, significantly reducing sleep-disturbing shoulder pain. Postoperative complications were minimal.

## Introduction

Shoulder pain and dysfunction can indicate rotator cuff tears (RCTs), primarily affecting the supraspinatus tendon.^1^ Approximately 30% of adults over 60 have some degree of RCTs.^2-4^ They usually occur because of traumatic injuries, degenerative changes, or a combination of both. However, there seems to be no distinction in degenerative alterations observed in ruptured tendons between patients with acute, trauma-related rotator cuff tears and those with non-traumatic, chronic tears.^5^ The RCTs can also progress as a long-lasting process, beginning with shoulder impingement that gradually gives rise to partial tears, full-thickness tears, massive tears, and eventually shoulder arthropathy.^2-4^ The degenerative tears typically affect the bursal surface or the main substance of the tendon. In contrast, younger athletes participating in overhead activities usually sustain tears at the articular surface secondary to repetitive tensile overload with subsequent fiber tension failure.^6^

The incidence of RCTs increases with age, partially because of age-related degeneration affecting the tendons and their attachments, and partly because of other possible associated comorbidities that can affect the quality of tendons and their response to overload (e.g., osteoporosis, diabetes mellitus, and rheumatoid arthritis).^7,8^ It is estimated that over 60% of people >80 years have RCTs, mostly asymptomatic despite being bilaterally affected.^9,10^ Progression to symptomatic tears is common within 5 years, mainly in patients with cuff muscle atrophy and fatty infiltration. Because of the growing aging population worldwide, RCTs are likely to be an increasing health care problem. Therefore, every effort should be made to improve their diagnostics and management. Fortunately, more advanced treatment strategies have been adopted over the past years due to an improved understanding of pathogenesis, diagnosis, patient selection, perioperative management, and surgical techniques.

However, there is still debate about the optimal treatment plan for older people with RCTs. Parameters such as tendon substance quality, retear risk, and functional demands and gains have been discussed as treatment outcomes. In their recently published systematic review,^11^ Hsieh et al found no significant differences between older and younger patients after arthroscopic repair of RCTs for functional improvement, retear rate, pain level improvement, muscle power, and shoulder range of motion. Nolte et al.^12^ and Altintas et al.^13^ found that healthy older patients without significant comorbidities who desire to return to an active lifestyle could benefit equally from arthroscopic repair as their younger counterparts. Chronological age did not seem to be an independent risk factor for failure; however, comorbidities associated with age may be. Therefore, these authors considered patient selection to be a crucial component dictating successful outcomes. However, there is a study that identified that older age, as an independent factor, contributes to an increased risk of healing failure after early arthroscopic repair in patients with trauma-related full-thickness rotator cuff tears.^14^ In clinical practice, older patients who do not respond well to non-surgical management are still good candidates for operative treatment, even without adequate evidence. Surgeons often choose to operate on these patients because it is difficult to ignore the long-term consequences of untreated symptomatic RCTs, which tend to increase in size and can give rise to shoulder rotator cuff tear arthropathy. Moreover, research indicates that two-thirds of patients with a mean age of 61 who undergo RCT repair exhibit successful healing.^15^ Notably, these patients experience superior outcomes compared to patients who do not achieve healing.^15^ Additionally, a study published in 2020 found that patients over 75 years old with symptomatic RCTs, who did not have advanced muscle degeneration at the time of surgery, reported good clinical outcomes and high patient satisfaction at midterm follow-up after undergoing arthroscopic rotator cuff repair.^16^

The present study aimed to evaluate the results of arthroscopic repair of RCTs in patients aged 60 years and older, to determine the effect of this surgical intervention on sleep-disturbing shoulder pain and range of motion.

## Materials and Method

### Study Design and Settings

We retrospectively included a case series of patients treated with arthroscopic repair of RCTs at a Hospital in Sweden, between 1 January 2018 and 1 January 2021.

### Patients

The study population was identified by searching for the patients’ international classification of disease codes in medical records. The inclusion criteria were patients ≥60 years old with MRI-verified RCTs with severe complaints, including reduced range of motion and sleep-disturbing shoulder pain. The patients should have undergone physiotherapy for at least 6 months and at least one subacromial corticosteroid injection. Patients under the age of 60, those whose procedures were converted from arthroscopic to open surgery, and individuals referred for urgent surgery due to severely reduced range of motion were excluded from the study.

**Figure 1.**
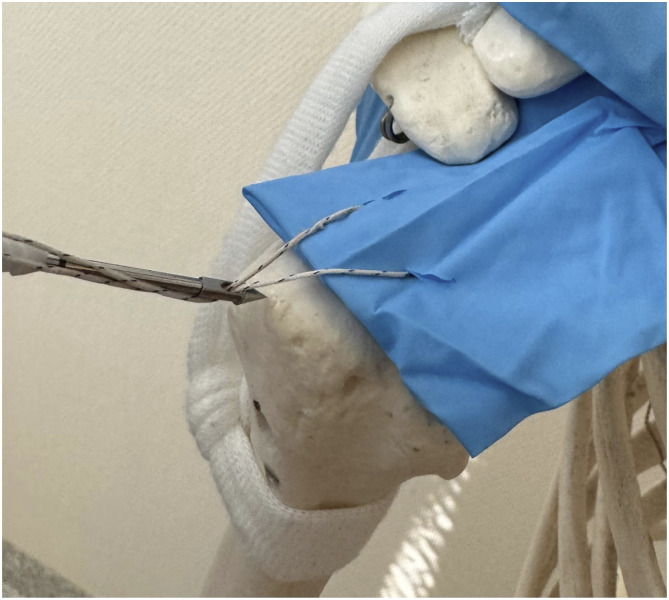
SpeedFix Suture technique.

**Figure 2.**
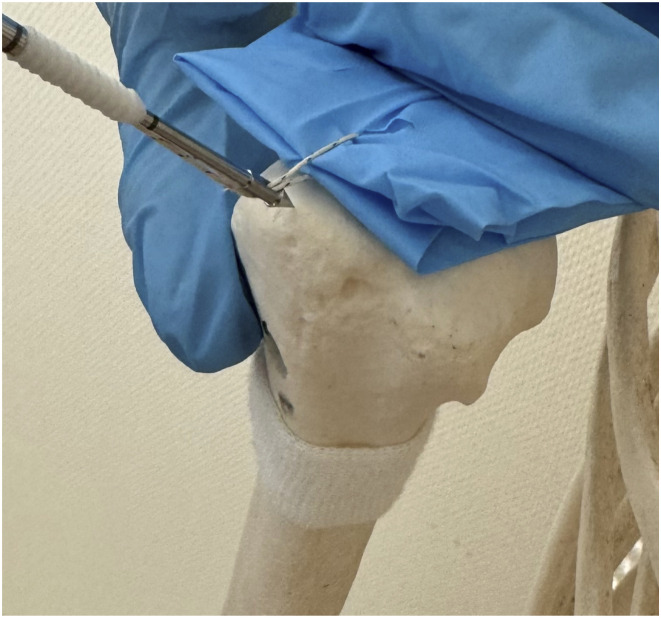
Single interrupted suture technique.

The arthroscopic RCT repair was chosen at the discretion of the treating surgeon. Arthroscopic repair employing a 4.5 mm PushLock suture anchor was conducted through posterior and lateral portals, using two distinct types of suture material — specifically Ethibond and Fibertape along with two suture techniques (Figure 1). The first technique is a speed-fix technique where a SCORPION-multifire suture passer is used to pass an inverted mattress stitch. The mattress stitch is then inserted into the anchor eyelet and implanted into the prepared bone socket, completing the knotless repair. The second technique is a single interrupted technique where a SCORPION-multifire suture passer is used to pass a single suture in one step. The stitch is then inserted into the anchor eyelet and implanted into the prepared bone socket, completing the knotless repair Figure 2.

The postoperative protocol includes the use of a shoulder abduction orthosis for 6 weeks. During this period, the rehabilitation regimen consists of the following: elbow exercises initiated on postoperative day one, pendulum shoulder exercises commenced one week postoperatively, passive shoulder exercises introduced three weeks postoperatively, and active shoulder exercises begun 6 weeks postoperatively. This structured approach ensures a gradual and controlled progression of shoulder rehabilitation to facilitate optimal recovery.

### Data Collection

Before the operation, we documented the age of patients, gender, laterality, details of RCT (site, extension, Tangent sign for muscle atrophy and eventual supraspinatus fatty infiltration on the sagittal T1-weighted MRI), and range of motion for flexion and abduction.

Operations were performed by one arthroscopic surgeon or directly under his supervision. Before the operation, one intravenous dose of 3 g Benzyl Penicillin and 2 g Cloxacillin was directly given as prophylaxis. We documented the number of tendons sutured and the anchors used.

After the operation, follow-up notes at 3 months were made by the treating surgeon and physiotherapist to document sleep-disturbing shoulder pain and range of motion for flexion and abduction. Postoperative complications were also recorded by searching the patients’ medical reports in May 2023, ie, 28 to 52 months after the operation. Postoperative MRI was conducted exclusively on patients who reported persistent pain and a lack of improvement in range of motion following the rehabilitation period, with the aim of detecting symptomatic re-ruptures.

### Statistical Analysis

A power analysis was performed using ClinCalc.com^17^ to determine the required sample size needed to evaluate the ability of the arthroscopic repair to reduce the incidence of sleep-disturbing shoulder pain from 100% before the operation to 60% after the operation. With a power of 0.90 and a significance level (alpha) of 0.05, a minimum of 40 patients would be needed. The material of 3 years of operative production (estimated to be more than 50 patients) was included to allow for drop-out.

Demographic data were analyzed by descriptive statistics and presented as means and standard deviations (SD). Comparisons between the pre- and postoperative parameters were performed by chi-squared tests for sleep-disturbing shoulder pain or dependent sample t-tests for the range of motion. Pearson’s correlation coefficient (*r*) was used to study the correlation between the patient’s age and improved range of motion before and after surgery.

All analyses were performed with R (version 4.2.2), knitr (version 1.41) for reproducible research, ggplot2 (version 3.4.0) for plots and Gmisc (version 3.0.1) with Greg (version 2.0.1) for table output. A *P*-value <0.05 was considered significant.

## Results

**Figure 3.**
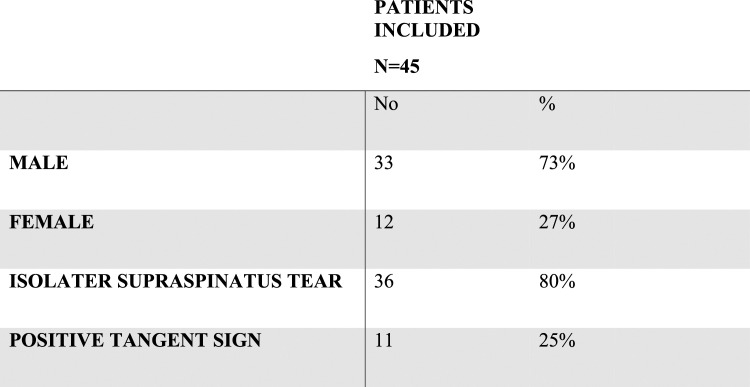
Study group data.

Fifty-three RCTs were treated with arthroscopic repair during the study period. Eight patients were excluded because of incomplete documentation, leaving a final sample of 45 (12 females, 27%) for enrollment (Figure 3). The patients’ mean age was 66 (range 60 to 76, SD 4.3) years. Almost 80% had isolated supraspinatus tears while 20% had a Collin type D tear.^18^ Twenty-five percent of patients had variable degrees of fatty infiltration on MRI examination (Goutallier grade 1-3) and a positive tangent sign. Only one patient had an MRI documented adhesive capsulitis.

During arthroscopy, tears were sutured using Ethibond or Fibertape. One-third of patients were treated with one anchor, whereas two-thirds required ≥2 anchors depending on the tear size. Also, the single interrupted suturing technique was used in one-third of the patients, while the speed-fix technique was used for the remaining patients. Almost 75% of patients underwent acromioplasty as part of the arthroscopic procedure.

We encountered no surgical site infections during the first three postoperative months. Symptomatic re-rupture, diagnosed with MRI, occurred in three patients (6%). At 3 months postoperative follow-up, 71% (n = 32) of patients had no sleep-disturbing shoulder pain (*P* < 0.001). Also, the mean abduction had improved from 62° (range 15 to 90, SD 25) to 122° (range 50 to 170, SD 36) (*P* < 0.001). The mean flexion had improved from 68° (range 15 to 150, SD 35) to 135° (range 70 to 170, SD 32) (*P* < 0.001).

The preoperative and postoperative assessments of active internal and external rotation were not thoroughly documented compared to abduction and flexion. This was mainly attributed to the patients' primary complaints being related to limitations in abduction and flexion. Data on external and internal rotation were available for 37 out of the 45 patients. The analysis revealed no significant changes in external rotation, with a preoperative mean of 66° (SD = 24°) and a postoperative mean of 60° (SD = 24°) (*P*-value = 0.363). Similarly, internal rotation showed no significant changes, with a preoperative mean of 95° (SD = 10°) and a postoperative mean of 95° (SD = 11°) (*P*-value = 0.99).

**Figure 4.**
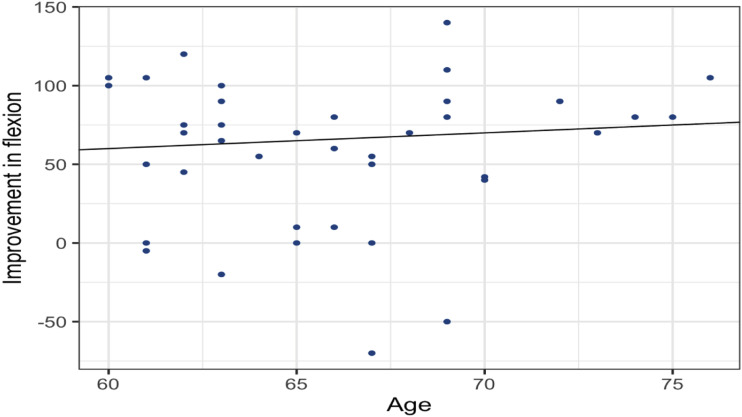
Pearson’s correlation coefficient (r) showing the correlation between the patient’s age and improvement of Flexion after surgery.

**Figure 5.**
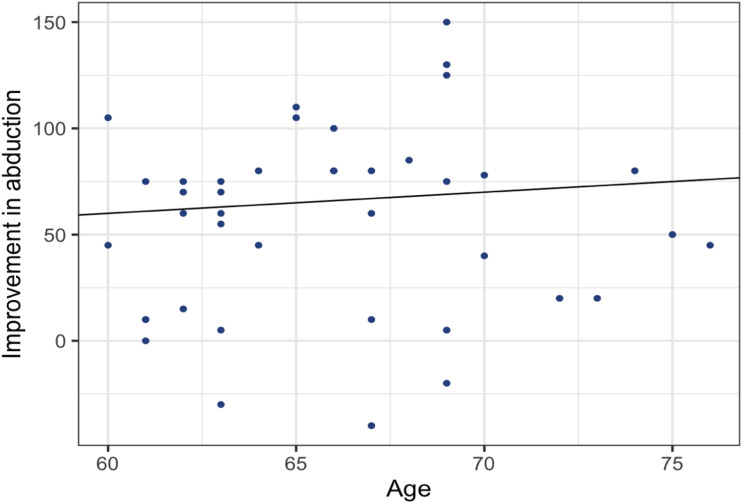
Pearson’s correlation coefficient (r) showing the correlation between the patient’s age and improvement in abduction after surgery.

We found a positive correlation between increasing age and postoperative improvement in flexion (r = 0.09, *P* = 0.60) and abduction (r = 0.07, *P* = 0.66). However, these findings were not significant (Figures 4 and 5).

## Discussion

The present study found that people aged 60 years and older who underwent arthroscopic repair of RCTs showed statistically significant clinical improvement in shoulder flexion and abduction, along with less sleep-disturbing shoulder pain. The slight reduction in postoperative external rotation (mean 6 degrees) can be attributed to the immobilizing post operatively. This difference was clinically and statistically insignificant. We encountered no surgical site infections, while only three cases (6%) had symptomatic re-rupture. These encouraging results may contribute to the existing literature, favoring the choice of surgical treatment for symptomatic RCTs in this age group with failed nonoperative treatment.

The reports in the literature about this issue are variable. For instance, Fabrizio et al^19^ conducted a meta-analysis that compared patients with RCTs surgical repair with conservative treatment. They could show that surgical treatment was associated with increased pain reduction and functional improvement at 6, 12, and 24 months. Even if these effects were often statistically significant, their clinical relevance was limited. Itoi and Tabata,^20^ on the other hand, studied 54 patients with RCTs who underwent conservative treatment, observing that 72% of individuals achieved good or excellent outcomes over an average follow-up period of 3.4 years. Samilson and Binder^21^ presented data of conservatively treated full-thickness rotator cuff tears (n = 292), revealing that 72% of shoulders achieved over 150° of abduction post-treatment, but 40% were deemed to have fair or poor outcomes. Bokor et al^22^ reported that 74% of patients with confirmed RCTs who received conservative management reported minimal or no pain after 7 years, with 86% expressing satisfaction with their outcomes. Therefore, patients are usually treated conservatively initially, leaving the surgical intervention to those who failed the conservative management.

During the past decade, surgeons have been enthusiastic about using arthroscopic repair of RCTs on a growing scale. The enthusiasm was fueled by various factors, including advancements in surgical techniques, suturing materials, and perioperative patient care. Moreover, older people presently maintain a more active lifestyle than in the past, which requires a management plan that promotes increased activity.^23^ In addition, the introduction of reverse shoulder arthroplasty as a salvage procedure for unsuccessful repairs may have encouraged surgeons to initiate an arthroscopic repair, even in more complicated RCTs.

The comparison of outcomes after arthroscopic repair of RCTs between young and older patients has been discussed in the literature. Hsieh et al conducted a systematic review and meta-analysis, examining 1244 papers. Among these, they included five non-randomized controlled trials of fair quality, which involved 671 participants (197 older patients and 474 younger patients). They found no significant differences between older and younger patients regarding improvement in Constant score, retear rate, or other outcomes (e.g., pain level improvement, muscle power, and shoulder range of motion).^11^ Conversely, Meng et al inquired about the feasibility of repairing RCTs in patients >75 years. They conducted a systematic review to find 249 shoulders operated arthroscopically with a significant improvement in clinical and functional scores (American Shoulder and Elbow Surgeons and Constant scores), postoperative pain, and patient satisfaction.^24^ Similar findings were reported by Fossati et al (2014) in a meta-analysis of 6 papers involving 504 shoulders in patients aged 70 and above.^25^ The incidence of postoperative retears reported by these reviews was higher (20% to 35%) than in our study (6%). This discrepancy could be due to the different methods used to diagnose the retears and the length of follow-up. Studies that prospectively collected data on retears with MRI reported a higher retear incidence, even though the impact of these retears in asymptomatic patients is questionable. Consistent with the present study, Stone et al^26^ documented a symptomatic retear incidence of 3.6% in their study population.^26^

Nocturnal pain is a common manifestation of RCTs and can lead to poor sleep quality and a decline in overall quality of life (QoL).^27,28^ Possible explanations for nocturnal pain include increased inflammatory reaction and mechanical pressure of the injured tendons, especially when sleeping on the affected side.^29^ In their systematic review, Barandiaran et al.^30^ studied this subject and identified 2198 RCTs with this complaint. They found that sleep quality was correlated with the amount of nocturnal pain but not shoulder function. In agreement with the present study, arthroscopic repair improved self-reported sleep in patients with shoulder pain. The review advised incorporating nocturnal pain and sleep quality as significant parameters in examining various treatment approaches for shoulder disorders, such as adhesive capsulitis and acromioclavicular joint diseases. In addition, Horneff et al. found that improvement in sleep quality after arthroscopic repair continued >2 years post-surgery, even though some patients in their study still had sleep disturbances.^31^

The current study has shortcomings. The retrospective design could compromise the amount and quality of our data. For example, there were no objective scores available regarding functional outcomes or QoL such as ASES or Constant scores and EQ-5D and the use subjective scores, such as the ASES, in future studies to assess functional outcomes in rotator cuff repairs, particularly in elderly patients is recommended. Also, only shoulder flexion and abduction were evaluated and documented thoroughly while information regarding internal and external rotation was partly available. We used the simple division of the presence or absence of sleep-disturbing shoulder pain without quantifying the severity of the pain. With our sample size, we think this division is adequate to serve the aim of the study, ie, to evaluate the effect of surgery on nocturnal pain. Furthermore, there was no control group to receive alternative treatment modalities. On the other hand, all patients had undergone previous nonoperative treatment without satisfactory results and were operated by the same orthopedic surgeon or under his direct supervision. This made the data more homogenous because technical variations and operative experience were not confounders. The operating surgeon was not part of this research study, thereby reducing potential interviewer bias.

## Conclusion

In conclusion, the arthroscopic repair of RCTs in patients aged ≥60 years is linked to significant improvement in shoulder flexion, abduction, and alleviation of sleep-disturbing shoulder pain in most patients 3 months after surgery. The occurrence of postoperative complications, such as surgical site infections and documented retears, was minimal. However, the limitations of this study design prevent drawing definitive conclusions, and further studies are warranted.
